# Deep Brain Stimulation for an Unusual Presentation of Myoclonus Dystonia Associated with Russell-Silver Syndrome

**DOI:** 10.5334/tohm.782

**Published:** 2023-10-30

**Authors:** Danielle S. Shpiner, Taylor K. Peabody, Corneliu C. Luca, Jonathan Jagid, Henry Moore

**Affiliations:** 1 Department of Neurology, University of Miami Miller School of Medicine, Miami, FL, USA; 2 Department of Neurological Surgery, University of Miami Miller School of Medicine, Miami, FL, USA

**Keywords:** uniparental disomy, myoclonus dystonia, Russell Silver syndrome, deep brain stimulation

## Abstract

**Background:**

Myoclonus dystonia syndrome typically results from autosomal dominant mutations in the epsilon-sarcoglycan gene (SGCE) via the paternally expressed allele on chromosome 7q21. There is evidence that deep brain stimulation (DBS) is beneficial for this genotype, however, there are few prior case reports on DBS for myoclonus dystonia syndrome secondary to other confirmed genetic etiologies.

**Case Report:**

A 20-year-old female with concomitant Russell-Silver syndrome and myoclonus dystonia syndrome secondary to maternal uniparental disomy of chromosome 7 (mUPD7) presented for medically refractory symptoms. She underwent DBS surgery targeting the bilateral globus pallidus interna with positive effects that persisted 16 months post-procedure.

**Discussion:**

We present a patient with the mUPD7 genotype for myoclonus dystonia syndrome who exhibited a similar, if not superior, response to DBS when compared to patients with other genotypes.

**Highlights:**

This report outlines the first described case of successful deep brain stimulation treatment for a rare genetic variant of myoclonus dystonia syndrome caused by uniparental disomy at chromosome 7. These findings may expand treatment options for patients with similar conditions.

Myoclonus dystonia syndrome is a condition that usually presents in childhood and is characterized by myoclonic jerks predominantly in the upper body. The associated dystonia typically manifests as torticollis or writer’s cramp but can occur in other areas of the body, or may be absent altogether, as in the isolated myoclonus phenotype [1]. Symptoms tend to be responsive to alcohol. Psychiatric symptomatology, including anxiety and depression, has also been reported. This condition is classically associated with autosomal dominant loss-of-function mutations in the epsilon-sarcoglycan gene (SGCE), a maternally imprinted (silenced) gene on chromosome 7q21.3 [2]. However, there are multiple described cases that are SGCE-negative and the myoclonus dystonia phenotype has further been linked to chromosomal region 18p11 and mutations in the *KCTD17, CACNA1B*, and *RELN* genes [3,4,5,6,7]. Rarely, it may result from maternal uniparental disomy of chromosome 7 (mUPD7), a mutation that is also associated with Russell-Silver syndrome [8,9].

Clinically, Russell-Silver syndrome is characterized by prenatal and postnatal growth restriction with resultant short stature and dysmorphic features such as relative macrocephaly, triangular face, clinodactyly, and café-au-lait spots on the trunk [8,9]. Molecular studies have identified abnormalities in chromosomes 7 and 11 as the most common etiologies, with up to 50% of cases related to methylation defects on chromosome 11p15, and roughly 10% to mUPD7 [10]. Those with mUPD7 may have a milder Russell-Silver syndrome phenotype and associated motor symptoms, which has previously been described in the literature [9,11].

While patients with medically-refractory myoclonus dystonia syndrome caused by SGCE mutation have been successfully treated with deep brain stimulation (DBS) of the globus pallidus pars interna (GPi), it has never been reported in a patient suffering from myoclonus dystonia syndrome associated with Russell-Silver syndrome secondary to mUPD7 [12,13,14,15,16,17,18,19,20,21].

## Case Report

We previously reported on a case involving an unusual association between myoclonus dystonia syndrome, Russell-Silver syndrome, and the mUPD7 variant [8]. In brief, a 20-year-old right-handed female with mUPD7, confirmed via PCR-based microsatellite marker analysis with absence of SGCE mutations, and features suggestive for Russell-Silver syndrome presented at age 18 for evaluation of abnormal movements. Her symptoms included onset of generalized body jerks at 13 years old with involvement of her head and all extremities. She described the movements as painful and non-suppressible, present at rest and with action. A few years later, she developed generalized dystonia, including subjective spasms in her lower extremities and frequent arching of her back, leading to frequent falls. Initial physical exam was significant for generalized, action-induced myoclonus and dystonic posturing affecting the neck, trunk, and extremities (Video 1). She had trialed multiple medications, including levetiracetam, topiramate, gabapentin, propranolol, clonazepam, and tetrabenazine, but symptoms persisted and severely limited her functional ability. She required assistance with multiple ADLs, particularly showering and going up/down stairs due to balance abnormalities. Due to fall risk, she had been using a walker since age 15 and progressed to a wheelchair by age 19.

**Video 1:** Patient exam at initial consultation. *Segment 1A*: The patient is seated in a chair. She is seen to have generalized myoclonus, worse with action, and dystonic posturing of the upper extremities, neck, and trunk. *Segment 1B*: The patient is walking down the hall; we see her ability to walk is limited by generalized myoclonus.

**Figure 1:**
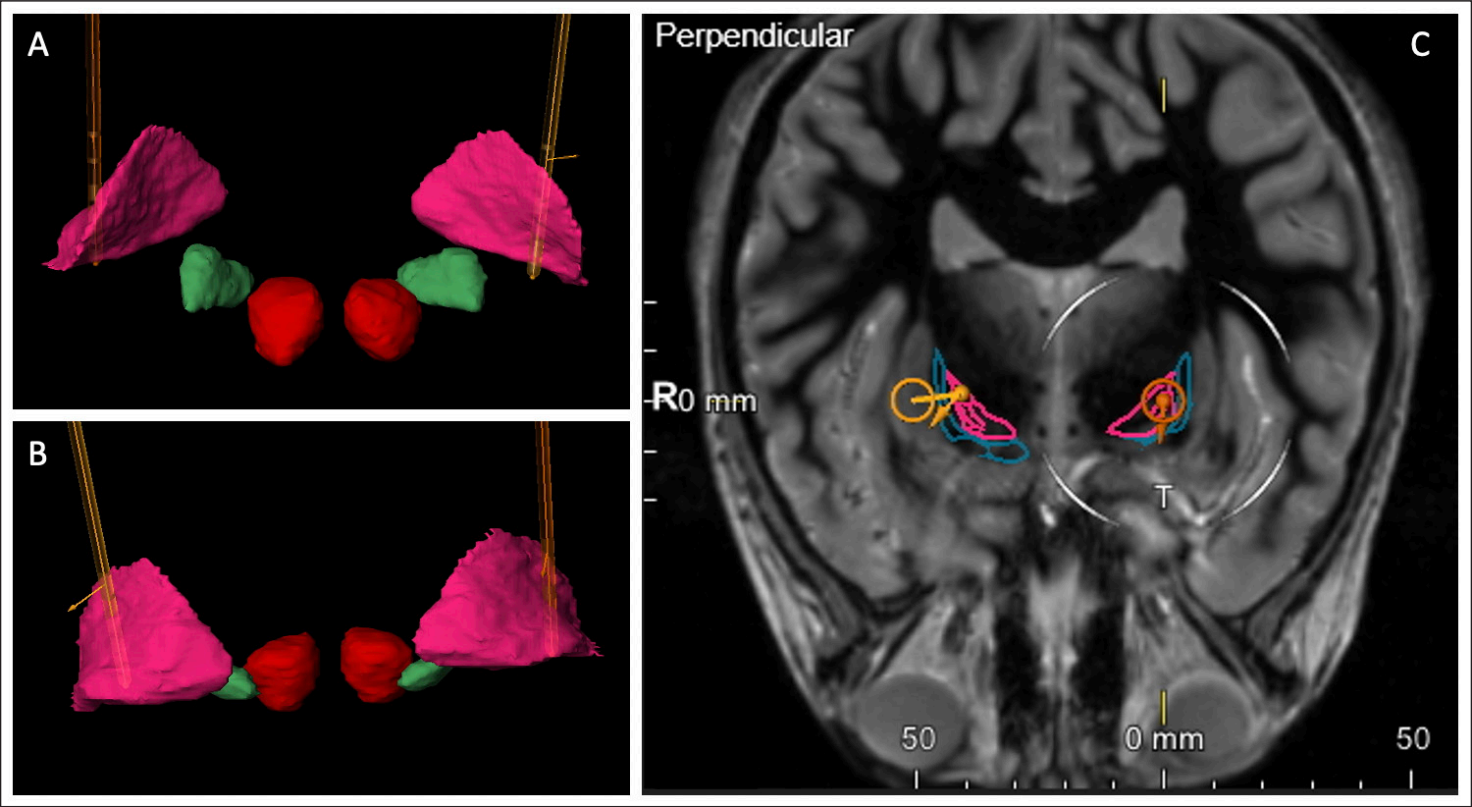
BrainLAB rendering of lead placement. **A:** Anterior view of lead placement in the bilateral GPi (pink). Subthalamic nuclei (green) and red nuclei (red) are illustrated for reference. **B:** Posterior view of lead placement. **C:** Coronal MRI reference imaging, which also highlights the bilateral globus pallidus externa (blue).

Given the incomplete response to medications and the significant disability produced by her symptoms, she underwent DBS surgery. The bilateral GPi were chosen as the target based on prior reports of successful DBS treatment for myoclonus dystonia syndrome [12,13,14,15,16,17,18,19,20]. BrainLAB software (BrainLAB Inc., Feldkirchen, Germany) was utilized for surgical planning and assisted in confirmation of lead placement (Figure 1). The procedure was performed using microelectrode recordings to map the borders of GPi and globus pallidus externa (GPe). GPe was mapped based on classical characteristics of intermittent low-frequency, low-density firing. GPi was identified when high amplitude, irregular firing was recorded. The microelectrode was advanced, and optic tracts were identified by inducing optic evoked potentials. Directional leads (Boston Scientific, Valencia, CA, USA) were ultimately placed with the bottom contacts at the inferior border of the mapped GPi. Macrostimulation was not performed due to patient being sedated and paralyzed. An implanted pulse generator (Vercise Gevia by Boston Scientific) was placed one week later. She then underwent a 2-week recovery period, after which, mapping and monopolar programming was performed. Intensity of stimulation was increased progressively up to the maximal tolerated setting, with a frequency of 130 Hz and a pulse width of 90 µs. Initial amplitude was 2 mA bilaterally, which has been increased to 4.3 mA since that time (Table 1).

**Table 1:** DBS Settings.


MONTHS POST-DBS	HEMISPHERE	CONTACTS	AMPLITUDE (mA)	PULSE WIDTH (µs)	FREQUENCY (Hz)

1	Left	C+, 2–, 3–, 4–	2	90	130

	Right	C+, 5–, 6–, 7–	2	90	130

31	Left	C+, 2–	4.3	60	130

	Right	3+, 6+, 2–, 4–, 5–, 7–	4.3	60	130


Changes in both her gait and myoclonus were appreciated as early as 3 weeks post-procedure, as seen in Video 2, and these symptoms progressively improved over the following 18 months. Nearly 2 years later, her myoclonus is mostly resolved, with mild dystonia appreciated at her lower extremities and trunk. Whereas she previously relied on a wheelchair, she is now able to ambulate briskly with a walker. We tracked her progress using multiple scales (Table 2), including the total Unified Myoclonus Rating Scale (UMRS) score, which improved from 208 pre-DBS to 14 at 6 months post-DBS; and Burke-Fahn-Marsden Dystonia Rating Scale (BFMDS) motor score, which decreased from 47 pre-DBS to 11 over the same time interval. Furthermore, we recorded her 36-Item Short Form Survey (SF-36) scores, which convey self-reported measures of health, to assess quality of life. Compared to her pre-DBS scores, her numbers were significantly increased at 6 months post-surgery, indicating improvement in her functional status. In terms of ADLs and IADLs, she cited improved ability to shower, eat, apply make-up, tie her shoes, brush her teeth, and get dressed without assistance post-DBS. Of note, her scores have remained largely stable at the 16-month mark with only slight worsening in her dystonia on the BFMDS motor scale.

**Video 2:** Patient exam 3 weeks post-DBS. *Segment 2A:* The patient is seated on an exam table. Her myoclonus is improved with various tasks in her upper extremities. *Segment 2B:* The patient is walking down the hall with a rolling walker. She is noted to have improvement in both her truncal dystonia and generalized myoclonus.

**Table 2:** **Pre- and Post- DBS Scores**. ^1^Comparison between pre-DBS scores and most recent values, ^2^Unified Myoclonus Rating Scale, ^3^Burke-Fahn-Marsden Dystonia Scale, ^4^36-Item Short Form Survey (each item scored 0–100).


SCALE/ITEM	PRE-DBS	POST-DBS (2 mo)	POST-DBS (6 mo)	POST-DBS (16 mo)	% IMPROVEMENT^1^

UMRS^2^					

Patient questionnaire	26	7	3	3	88.5

Patient global assessment	3	2	1	1	66.7

Myoclonus at rest	70	5	2	2	97.1

Stimulus sensitivity	12	0	0	0	100.0

Myoclonus action	78	5	2	2	97.4

Functional tests	12	5	4	4	66.7

Global disability	3	2	2	2	33.3

Negative myoclonus	1	0	0	0	100.0

Negative myoclonus severity	3	0	0	0	100.0

TOTAL (0–369)	208	26	14	14	93.3

**BFMDS MOTOR^3^**					

Eyes	4	0	0	0	100.0

Mouth	0	0	0	0	–

Speech & swallowing	0	0	0	0	–

Neck	6	4	0	0	100.0

RUE	4	1	1	1	75.0

LUE	6	1	1	1	83.3

RLE	6	1	3	3	50.0

LLE	9	1	0	3	66.7

Trunk	12	9	2	3	75.0

TOTAL (0–120)	47	17	7	11	76.6

**SF-36^4^**					

Physical functioning	5		55		50.0

Role limitations – physical	0		100		100.0

Role limitations – emotional	0		100		100.0

Energy/fatigue	35		60		25.0

Emotional well-being	24		56		32.0

Social functioning	12.5		100		87.5

Pain	22.5		90		67.5

General health	40		75		35.0


## Discussion

Several studies have shown responsiveness of myoclonus dystonia syndrome to DBS, even in patients presenting with isolated myoclonus phenotype or absent SGCE mutation [14,22,23,24]. Although GPi is more commonly selected as the target for this condition, DBS to the ventral intermediate thalamic nucleus (VIM) and even combined GPi + VIM has been reported [20]. Stimulation to VIM demonstrated a greater effect on myoclonus than dystonia with some studies showing a direct relationship between increased frequency of stimulation and improved myoclonus [21,24]. DBS to GPi has shown similar effect on myoclonus and generally superior treatment of dystonia when compared to VIM [13,20]. That said, studies are limited due to the rarity of myoclonus dystonia syndrome and optimal target has not yet been defined.

We selected GPi for our patient due to her generalized dystonia on presentation and had overall positive results. Her UMRS score indicated a 93.3% improvement in her myoclonus at 16 months post-surgery, with 76.6% improvement in dystonia per her BFMDS Motor score. These findings are similar to a case series by Azoulay-Zyss et al, which studied the effect of bilateral GPi DBS on five patients with SGCE-related myoclonus dystonia syndrome. This study had a similar follow-up period of 15–18 months and cited 83% and 85% improvement in myoclonus and dystonia, respectively [16]. Similarly, Kotsutzka et al. followed nine post-GPi DBS patients with SGCE mutations and noted mean improvement of 94.1% and 71.4% over an average follow-up of 8.7 years [12]. Other case series have reported slightly more modest improvements, particularly for dystonia [13,17,18,24]. For example, one of the larger studies by Gruber et al. reported an average 65.5% improvement in UMRS scores and 48.2% improvement in BFMDS scores for ten patients [15]. Rughani et al. observed greater effect on both myoclonus and dystonia with younger age at time of surgery and shorter duration between diagnosis and surgery, which may partially explain the higher percentage of improvement in our patient who had surgery at 21 years old (compared to their cohort which ranged from 24–69 years old) [13]. Given the limitations of a single case report, it is unclear whether the mUPD7 genotype influences the degree of improvement following DBS when compared to other genotypes.

With regards to stimulation parameters, our final pulse width (60 µs) and frequency (130 Hz) appear similar to that of other myoclonus dystonia syndrome cases with DBS to GPi. Our patient has benefitted from a monopolar configuration on the left and bipolar configuration on the right, the latter to minimize side effects of paresthesias (Table 2). It is important to note that, in general, the DBS effects on myoclonus are long-lasting, whereas the effects on dystonia seem to diminish over time, which is also reflected in our patient [19]. As such, the amplitude of stimulation has been increased over time to manage her dystonia.

To our knowledge, this is the first described case of successful DBS for medically-refractory myoclonus-dystonia syndrome associated with Russell-Silver syndrome caused specifically by the mUPD7 variant. Our patient showed significant improvement in both objective measures of myoclonus and dystonia, and in self-reported quality of life after surgery. At 16 months post-surgery, the improvements in myoclonus were sustained, with mild worsening in her dystonia as compared to 6 months post-surgery. Her overall functional status remains improved. These results suggest that DBS may be a good option for other patients with medically-refractory myoclonus dystonia syndrome and the mUPD7 genotype.
